# Dual Pathology: A Rare Association in Surgical Acute Abdomen

**DOI:** 10.7759/cureus.15926

**Published:** 2021-06-25

**Authors:** Ravi Gupta, Samiksha Parashar, Richa Gupta

**Affiliations:** 1 General Surgery, All India Institute of Medical Sciences, Gorakhpur, IND; 2 Anesthesia and Critical Care, Dr. Ram Manohar Lohia Institute of Medical Sciences, Lucknow, IND; 3 Ophthalmology, King George's Medical University, Lucknow, IND

**Keywords:** dual pathology, acute abdomen, gastric perforation, acute mesenteric ischemia, small bowel ischemia

## Abstract

Among the causes of acute surgical abdomen intestinal perforation and mesenteric ischemia are some of the leading causes of morbidity and mortality. Gastric perforation has a mortality rate of 20-30% and mesenteric ischemia has a mortality of 60%. The presence of both pathologies in the same patient at the same time is a rare association and very few cases have been reported till now. This association has been described in cases of polyarteritis nodosa. Here we are presenting our case in which no such vascular pathology was identified. We have tried to hypothesize the reason behind this rare association.

## Introduction

Mesenteric ischemia defines insufficient mesenteric perfusion to fulfill the metabolic demand. They are classified as acute mesenteric ischemia (AMI) and chronic mesenteric ischemia (CMI). Clinical presentations are nonspecific, so prompt diagnosis and early management are needed in these cases as they have a high mortality rate of 24%-94% [1].

AMI can be classified into four types according to the cause. Previously arterial emboli were the commonest cause but recent studies have shown that arterial thrombosis is the most common cause of AMI constituting 50% of arterial AMI [2,3]. The superior mesenteric artery (SMA) is the most common site for arterial AMI due to its high flow state and acute angle origin [4]. The next most common cause is non-occlusive mesenteric ischemia (NOMI) which is associated with a low cardiac output state and diffuse splanchnic vasoconstriction due to hypotension or vasopressors [5,6]. The least common is mesenteric vein thrombosis (MVT) and constitutes 2.9%-15% of all AMI. The superior mesenteric vein is most commonly involved and mortality is less than arterial AMI [7]. MVT involvement of bowel is usually segmental and transition time to become a bowel ischemic is longer than arterial AMI. 

Perforation due to peptic diseases has become less common due to antacids and proton pump inhibitors. The incidence of perforation in gastric ulcers is less nowadays but is associated with a high mortality rate of 20%-30% [8]. As both perforation and mesenteric ischemia have a high morbidity and mortality rate. Vascular diseases like polyarteritis nodosa and atherosclerosis can present both above pathologies in the same patient at the same time. Though very few cases have been reported till now, here we are presenting a similar case and are trying to hypothesize the cause of the association.

## Case presentation

**Figure 1:**
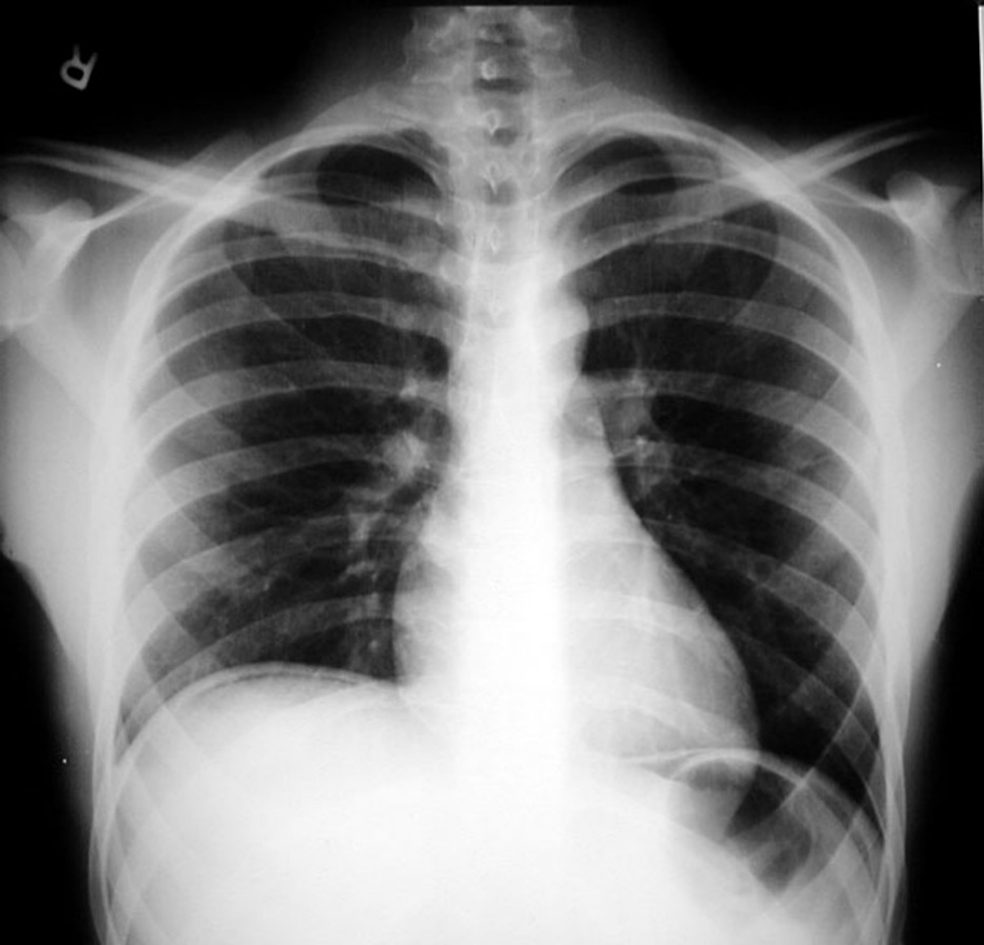
X-ray chest showing free gas under right hemidiaphragm.

A 40-year-old gentleman, a chronic smoker, presented to the ED with acute pain in the upper abdomen for one day. The pain was associated with abdominal distension, fever, and obstipation. He had no associated comorbidities related to diabetes mellitus or hypertension and no history of long-term intake of analgesics like nonsteroidal anti-inflammatory drugs (NSAIDs). There was no history of pain of a similar nature. On examination, there was tachycardia of 110/min, tachypnoea of 25/min, and generalized abdominal guarding was present. Blood investigations showed a leucocytosis of 23000 cells/mm^3^ with neutrophil predominance, serum creatinine was on the slightly higher side that is 2 mg/dl, serum amylase was raised, arterial blood gas analysis was suggestive of metabolic acidosis with pH of 7.1, and serum lactate was raised by 10 mmol/L. X-ray chest showed free gas under the right side of the diaphragm suggestive of pneumoperitoneum due to intestinal perforation (Figure 1).

**Figure 2:**
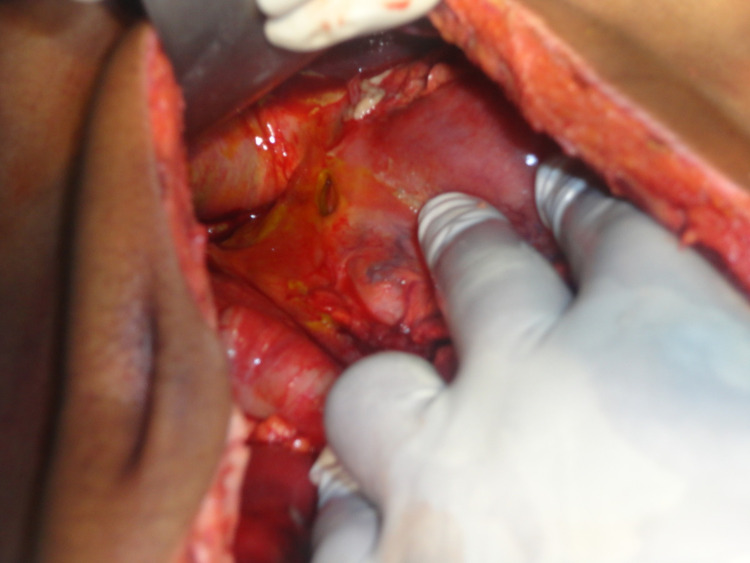
Intraop picture showing gastric prepyloric perforation.

**Figure 3:**
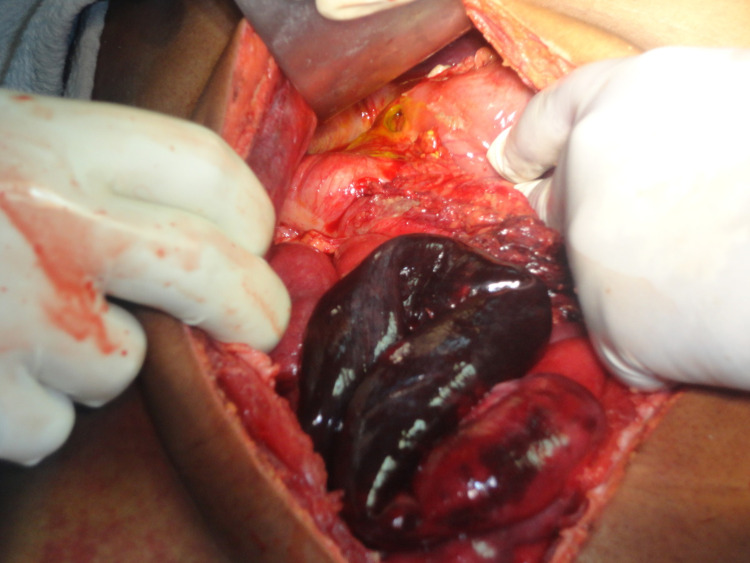
Intra op picture showing gastric perforation along with ischemic small bowel.

**Figure 4:**
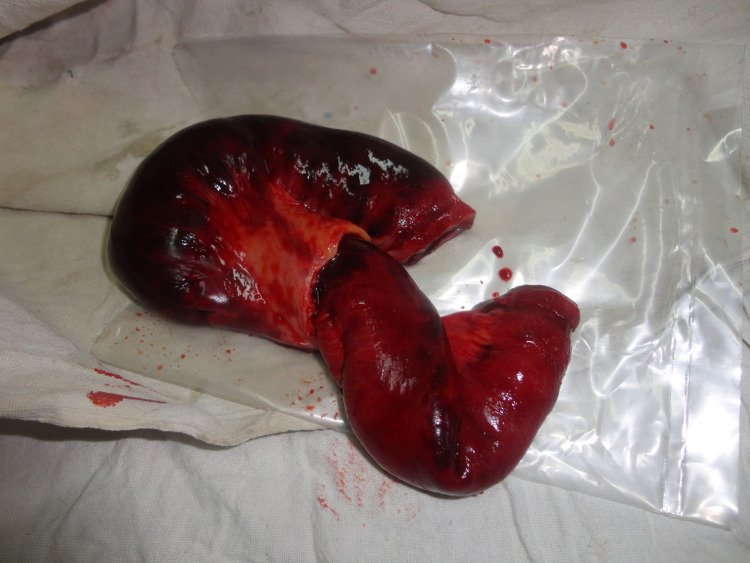
Resected ischemic small bowel specimen.

The patient had a urine output of around 0.5 ml/kg/min. He was resuscitated for four hours with intravenous (IV) fluid and an attempt was made to correct metabolic acidosis. Further, he was taken for emergency laparotomy. Intraoperatively, there was prepyloric gastric perforation (Figure 2). Along with that, there was approximately one-foot gangrenous distal ileum, two feet proximal to the ileocecal junction (Figure 3). We performed a biopsy of the ulcer edge with omental patching along with resection of a gangrenous segment of the distal small bowel (Figure 4) with double barrel ileostomy. The post-operative period was uneventful except till the 4th postop day when the patient was on noninvasive ventilatory support and on a postop day 10 when the patient was discharged. Histopathology of resected small bowel specimen showed normal mesenteric vessels with ischemic changes in submucosa and muscle layer. Closure of ileostomy was done after three months.

## Discussion

On reviewing the literature, we found two case reports on a patient presenting as an acute abdomen having dual pathology of gastric perforation and mesenteric ischemia at the same time [9,10]. By two hypotheses, the association between two pathologies can be justified. The first view, if it all disease started with gastric perforation, then due to peritonitis and hypotension, AMI can occur in a patient with normal vessels due to splanchnic vasoconstriction or atherosclerotic one due to arterial thrombosis. As in our case, the patient was a chronic smoker, which predisposed him for the development of gastric perforation first followed by AMI. Further, chronic smokers are also at risk of atherosclerosis which probably had initiated thrombus formation in the mesenteric vessel in the acute surgical abdomen in our case. The second view is if the patient is having a vascular disease like polyarteritis nodosa, can also present with dual pathology at the same time [9]. In our case, histopathology of resected bowel showed no feature suggestive of polyarteritis nodosa, so we assume that the first reason can be a way to justify this dual pathology in our patient.

## Conclusions

Gastric perforation with AMI in the patient with acute abdomen is a rare association and it should be evaluated for vascular diseases. Due to poor general conditions at the time of presentation, early diagnosis and damage control surgical management should be preferred.
